# Unilateral follicular variant of papillary thyroid carcinoma with unique *KRAS* mutation in struma ovarii in bilateral ovarian teratoma: a rare case report

**DOI:** 10.1186/1471-2407-12-224

**Published:** 2012-06-08

**Authors:** Boban Stanojevic, Radan Dzodic, Vladimir Saenko, Zorka Milovanovic, Vesna Krstevski, Petar Radlovic, Marko Buta, Bozidar Rulic, Lidija Todorovic, Bogomir Dimitrijevic, Shunichi Yamashita

**Affiliations:** 1 Nagasaki University Graduate School of Biomedical Sciences, 1-12-4 Sakamoto, Nagasaki, 852-8523, Japan; 2 Laboratory for Radiobiology and Molecular Genetics “Vinca”, Institute of Nuclear Sciences, University of Belgrade, Mike Petrovica Alasa12-14, P.O. Box 522, 11000, Belgrade, Serbia; 3 University of Belgrade, School of Medicine, Dr Subotica 8, 11000, Belgrade, Serbia; 4 Institute of Oncology and Radiology of Serbia, Surgical Oncology Clinic, Pasterova 14, 11000, Belgrade, Serbia; 5 Department of Pathology, Institute for Oncology and Radiology of Serbia, Pasterova 14, 11000, Belgrade, Serbia; 6 Department of Pathology, Medical Center Valjevo, 14000, Valjevo, Serbia

**Keywords:** Struma ovarii, Follicular variant of papillary thyroid carcinoma, *KRAS* mutation

## Abstract

**Background:**

Struma ovarii (SO) is a rare form of ovarian mature teratoma in which thyroid tissue is the predominant element. Because of its rarity, the differential diagnosis between benign and malignant SO has not been clearly defined. It is believed that malignant transformation of SO has similar molecular features with and its prognosis corresponds to that of malignant tumors originating in the thyroid.

**Case presentation:**

We report 35-year-old woman with bilateral ovarian cysts incidentally detected by ultrasound during the first trimester of pregnancy. Four months after delivery of a healthy child without complication she was admitted to the hospital for acute abdominal pain. Laparoscopic left adnexectomy was performed initially in a regional hospital; right cystectomy was done later in a specialized clinic. Intraoperative frozen section and a final pathology revealed that the cyst from the left ovary was composed of mature teratomatous elements, normal thyroid tissue (>50%) and a non-encapsulated focus of follicular variant of papillary thyroid carcinoma (PTC).

Normal and cancerous thyroid tissues were tested for *BRAF* and *RAS* mutations by direct sequencing, and for *RET/PTC* rearrangements by RT-PCR/Southern blotting. A *KRAS* codon 12 mutation, the GGT → GTT transversion, corresponding to the Gly → Val amino acid change was identified in the absence of other genetic alterations commonly found in PTC.

**Conclusion:**

To the best of our knowledge, this is the first time this mutation is described in a papillary thyroid carcinoma arising in struma in the ovarii. This finding provides further evidence that even rare mutations specific for PTC may occur in such tumors. Molecular testing may be a useful adjunct to common differential diagnostic methods of thyroid malignancy in SO.

## Background

Struma ovarii (SO) is a rare form of ovarian teratoma where thyroid tissue forms a grossly detectible mass or is the major cellular component (*>*50%). Teratomas comprise approximately 95% of germ cell tumors of which SO accounts for only 3% [1,2]. The differential diagnosis between benign and malignant SO may be difficult due to the lack of uniform diagnostic criteria [3]. The real incidence of malignancy in SO is unclear because of its rarity but the existing literature indicates that 5%–10% of such tumors are malignant with papillary and less frequently, follicular carcinoma being the most common [1-4]. It is believed that malignant transformation of SO has similar molecular features with and its prognosis corresponds to that of malignant tumors originating in the thyroid [5,6]. The molecular pathogenesis of papillary thyroid carcinomas (PTC) is largely associated with genetic alterations in the RET-RAS-RAF-MAPK pathway usually caused by *RET/PTC* rearrangements (13%–43%) or activating point mutations in the *BRAF* (29%–83%) or *RAS-*family genes (0 %–21 %). These molecular alterations occur cumulatively in up to 70% of all follicular cell-derived thyroid carcinomas in a nearly mutually exclusive manner [6-9].

## Case presentation

**Figure 1:**
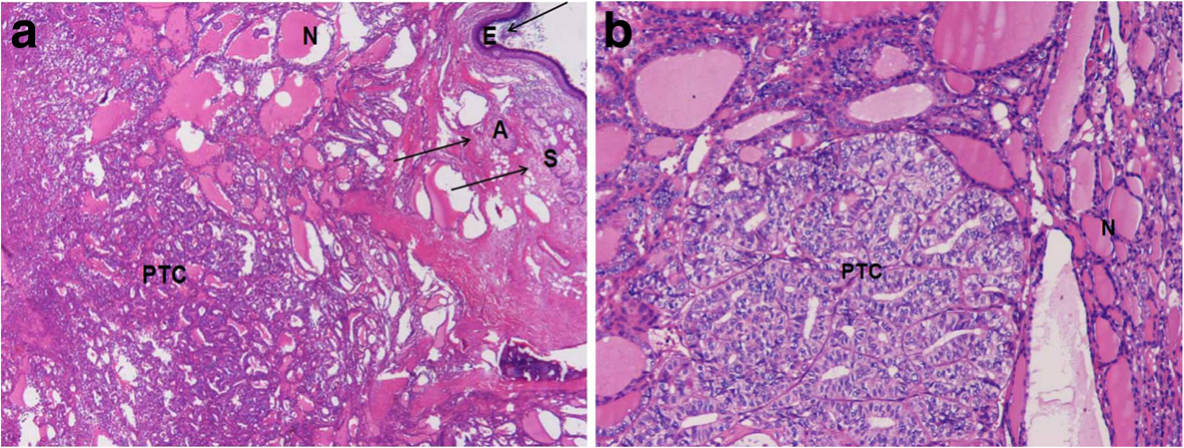
**a. Teratoma tissue with mature epidermis (E), sebaceous glands (S), adipose tissue (A) normal (N) and malignant thyroid tissue (PTC) (H&E 40x). b.** Fragment of PTC in SO featuring solid-trabecular and follicular growth patterns (H&E 100x).

The patient is a 35-year-old woman (gravida 1, para 0, abortus 0) who during the first trimester of pregnancy was diagnosed for bilateral ovarian cysts detected incidentally by ultrasound. She had no significant medical or gynecological history before and was clinically and biochemically euthyroid with normal serum TSH level. Four months after delivery of a healthy child without a complication she was admitted to the hospital for acute abdominal pain. Laboratory data showed normal biochemical parameters and complete blood count. The plasma level of CA-125 was slightly elevated to 42.72 U/ml (35 U/mL, the upper normal limit). An ultrasound scan showed an enlarged left ovary and cystic masses in both ovaries. The patient was operated in December 2008 in a regional hospital where laparoscopic left adnexectomy was initially performed. Intraoperative frozen section pathology revealed that the cyst was composed of mature teratomatous elements including epidermis, sebaceous glands, adipose, normal thyroid tissue (>50%) and a 10 mm non-encapsulated cancerous focus (which was confirmed later to be the FV-PTC on final pathology, Figure 1a and 1b) [10-12]. Because of the finding of cancer the surgeon made a decision not to proceed with the right oophorectomy and to transfer the patient to specialized oncology clinic.

**Figure 2:**
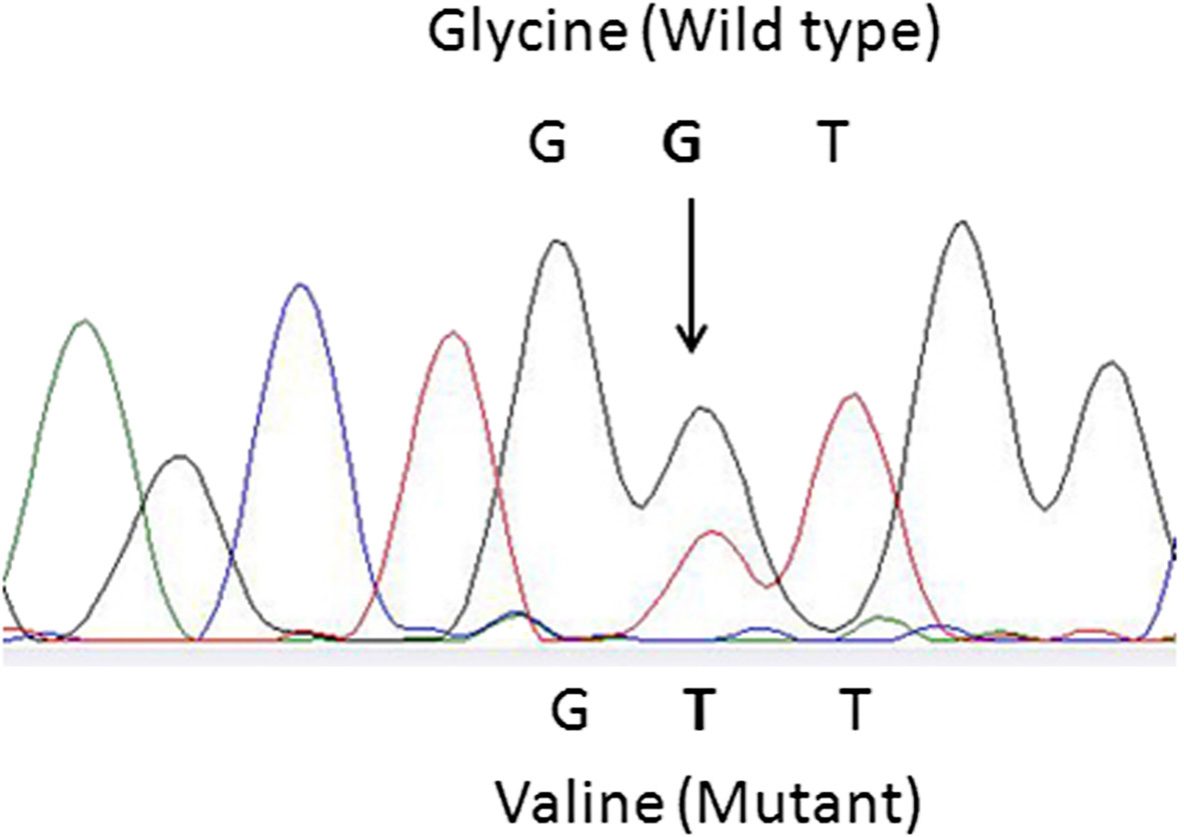
***KRAS*codon 12 mutation, GGT → GTT, corresponding to the Gly → Val amino acid change.**

The tumor from the left ovary showed immunoreactivity to thyroglobulin and thyroid transcription factor-1 (all antibodies from Dako, Carpinteria, CA, USA, data not shown). For molecular investigation 5 μm sections were taken, dewaxed, and lightly stained with hematoxylin and eosin. Normal thyroid tissue and PTC area were manually dissected from 3 serial sections. DNA was extracted using Gentra Puregene Tissue kit (Quiagen, Minneapolis, MN, USA). Total RNA was extracted using Recover All Total Nucleic Acid Isolation Kit *(*Ambion, Applied Biosystems, Foster City, CA, USA). DNA from the tumor and adjacent benign thyroid tissue was tested by PCR/direct sequencing of genomic DNA for genetic abnormalities that have been described in thyroid cancer originating in the neck, including a portion of *BRAF* exon 15 and codons 12, 13, 31, 60 and 61 of *K-H-* and *N-RAS. RET/PTC1* and *RET/PTC3* rearrangements were detected using reverse transcription-polymerase chain reaction (RT-PCR) followed by Southern blotting. This tumor was found to harbor a *KRAS* codon 12 mutation, the GGT → GTT transversion, corresponding to the Gly → Val amino acid change (Figure 2). No mutations were found in adjacent benign thyroid tissue. All experimental techniques and primer sequences used were essentially as previously described [13].

Case was presented to the multidisciplinary committee at the Institute of Oncology and Radiology of Serbia who decided the patient should undergo reoperation for the right ovarian cyst measuring 80 mm, which was also present at the time of the first surgery. The patient was operated two months after the initial surgery and after median laparatomy a macroscopically enlarged right ovary was verified to contain two cysts. The larger cyst measured 50x45x25 mm and contained soft whitish material. Histology report confirmed benign ovarian cyst with no evidence of other abnormality. An intra-operative frozen section and final pathology of the second smaller cyst 30x25x20 mm in size revealed mature cystic teratoma with benign thyroid tissue. Other tissue components included hair, adipose tissue, cartilage, bone and skin. Both cysts were removed and the right ovary was preserved. Multiple biopsies of the greater omentum, iliac lymph nodes, peritoneum above the urinary bladder and cul de sac showed no signs of malignant spread. Postoperative thyroid function blood test showed normal TSH, calcitonin, thyroglobulin and thyroglobulin antibody levels. Neck ultrasonography revealed a solitary thyroid nodule (6x4 mm) located caudally in the left lobe without signs of enlarged central and jugulo-carotid lymph nodes. No evidence of distant metastases was found on abdominal ultrasonography and chest X-ray. The patient is currently well, without evidence of disease progression one year after the second surgery. She is closely followed up undergoing clinical, radiologic and laboratory examinations every 6 months. At present, the patient is planned for fine needle aspiration biopsy; total thyroidectomy is considered as a further treatment modality [14].

## Conclusion

Thyroid-type carcinoma in SO is rare. Only 6% of cases of malignant transformation in struma ovarii are bilateral and the tumors arise more commonly in the left ovary than in the right [1,2,4]. In the described case the tumor was unilateral on the left side. Due to the rarity of the tumor, the real incidence of malignancy, pathobiology and molecular profile of thyroid cancer arising in the ovary remain poorly studied.

Thus far only a few studies have analyzed genetic alterations in thyroid-type carcinoma in SO. Previous case series describe the presence of point mutations in *BRAF, NRAS, HRAS*, and *RET/PTC* rearrangements but none in the *KRAS* gene [6,15-17]. The present report therefore describes for the first time a *KRAS* codon 12 mutation in a case of PTC with a follicular growth pattern arising in SO in combination with bilateral teratoma [10-12].

The prevalence of *RAS* gene family mutations in thyroid-originating PTC ranges from 0 to 21% in different series (about 11% on average) [18,19]. The highest frequency of *RAS* mutations has been found in the follicular variant of PTC (43%) while in the tumors with other morphology they are not so common [20]. Alterations most frequently involve codon 61 of *HRAS* and *NRAS* and less frequently codons 12 and 13; mutations of *KRAS* are very rare in general [21-23]. In line with these observations, our previous study in Serbian patients detected *RAS* mutations in 6/218 (2.8%) classical papillary tumors and 5/44 (11.4%) follicular variant PTCs. Most detected mutations were in codon 61 of *NRAS* (8/11, 72.7%) and 3/11 (17.3%) were in codon 12 of *KRAS*[13].

Thyroid tumors with mutant *RAS* are frequently encapsulated and display a lower rate of nodal disease which are favorable prognostic factors. On the other hand, *RAS* mutations are also found in approximately one-half of poorly differentiated and anaplastic thyroid carcinomas and are associated with poor patient survival, suggesting that *RAS* may have distinct roles in the early and late stage of thyroid cancer [19,20,24]. Several studies have demonstrated that tumors carrying any of mutant forms of *KRAS* are associated with a poor prognosis in a number of human malignancies [24,25]. Analyzing these reports, we found that a glycine to valine mutation at codon 12 of the *KRAS* gene, the same mutation that we describe in this report, associates with poor survival in colon cancer [25] suggesting that this mutation may represent a marker of tumor aggressiveness, perhaps in thyroid cancer as well.

On the other hand*, BRAF* mutations or *RET/PTC* rearrangements are commonly seen in thyroid cancer arising in struma ovarii. In one of the largest and most significant studies to date, Schmidt et al., showed that every PTC in SO with the classical growth pattern had the *BRAF*^*V600E*^ mutation, and the two mutant *BRAF*-harboring tumors with follicular architecture had *BRAF*^*K601E*^ and *BRAF*^*TV599–600M*^ mutation. No follicular carcinomas were identified in this study. Genetic alteration in benign SO has been seen in only one case in the literature. Elisei et al. recently reported the presence of *RET*/*PTC3* rearrangement in histologically benign tissue. In carcinomas of the thyroid, numerous studies have suggested, although equivocally, that *BRAF* mutation associates with the older age, advanced disease, classical papillary histology and poorer prognosis as indicated by disease-free and overall survival [8,9]. The presence of *RET/PTC* rearrangement correlates with some clinicopathological features of PTC such as younger age of patients, tumor morphology and a higher probability of lymph node involvement [26]. No studies have as yet correlated *RAS* and *BRAF* mutational status or the presence of *RET/PTC* rearrangements with clinical behavior in thyroid-type carcinoma arising in SO. However, it is generally assumed that clinical behavior and prognosis of these tumors are similar to those of tumors arising in the thyroid [6,15-17].

In our study, we describe an unusual case of KRAS mutation-harboring FV-PTC in a patient with bilateral ovarian teratoma. This mutation has not been previously reported in any SO, although it is sometimes detected in PTC of the thyroid and in a variety of other tumor types. Analysis of more cases and a longer period of follow-up will be necessary to determine whether this mutation has prognostic or therapeutic value.

## Consent

Written informed consent was obtained from the patient for publication of this case report and any accompanying images. A copy of the written consent is available for review by the Editor-in-Chief of this journal.

## Abbreviations

SO: Struma ovarii; PTC: Papillary thyroid carcinoma; TSH: Thyroid - stimulating hormone; CA-125: Cancer antigen; PCR: Polymerase chain reaction; FV-PTC: Follicular variant of papillary thyroid carcinoma; Gly: Glycine amino acid; Val: Valine amino acid.

## Competing interests

We declare no conflict of interests.

## Authors’ contributions

The patient was examined and operated by PR and BR and these authors are responsible for the post-operative care, follow-up and clinical information. RD and MB examined the patient and reviewed the patient’s files. RD will operate the patient. ZM and VK performed histopathological examination. BS and VS were responsible for the main conception and laboratory investigation. The manuscript was drafted by BS and VS, and then critically reviewed by LT, BD and SY. All authors read and approved the final manuscript.

## Pre-publication history

The pre-publication history for this paper can be accessed here:

http://www.biomedcentral.com/1471-2407/12/224/prepub
